# Clinical, humanistic, and economic outcomes of *Passiflora alata* Curtis use in participants with mild to moderate anxiety: a non-randomized experimental study

**DOI:** 10.1007/s40199-026-00591-4

**Published:** 2026-02-04

**Authors:** Thatiane Bárbara de Barros, Astrid Wiens, Tiago Marques dos Reis

**Affiliations:** 1 Federal University of Alfenas (UNIFAL-MG), Alfenas, Brazil; 2 Federal University of Paraná (UFPR), Curitiba, Brazil

**Keywords:** Anxiety, Herbal medicine, Passiflora, Pharmaceutical care, SUS

## Abstract

**Background:**

Anxiety is a disorder treated through psychotherapy and/or pharmacotherapy. *Passiflora alata* Curtis is a herbal medicine used as a therapeutic alternative, although clinical evidence supporting its effectiveness in anxiety management is limited.

**Objectives:**

This study aimed to evaluate the clinical, humanistic, and economic outcomes in participants with anxiety treated with *P. alata* extract.

**Methods:**

This was an open-label, non-randomized, before-and-after experimental study. Inclusion criteria were: age ≥ 18 years and anxiety symptoms. The clinical outcome was assessed using the GAD-7 scale, comparing scores between baseline (t0) and the final follow-up (t3). The humanistic outcome was assessed using the TSQM, applied 15 days after treatment initiation and again at t3. The economic outcome consisted of analyzing direct treatment-related costs.

**Results:**

*P. alata* demonstrated clinical effectiveness in 29 participants (96.7%). TSQM results indicated a perceived improvement in health status within the first weeks. Regarding economic outcomes, differences were observed between the protocols that included *P. alata* and the conventional treatments offered by the Brazilian Unified Health System.

**Conclusion:**

It was concluded that the use of *P. alata* extract for anxiety management is a potential therapeutic alternative for incorporation into the Brazilian Unified Health System.

**Supplementary Information:**

The online version contains supplementary material available at 10.1007/s40199-026-00591-4.

## Introduction

Anxiety is recognized as one of the leading causes of disability in Western countries, significantly impairing quality of life [1]. In Brazil, it presents a high prevalence, particularly among urban workers [2], and may become dysfunctional when exacerbated [3]. Treatment typically involves psychotherapy and/or pharmacotherapy, including over-the-counter options in mild cases. Among these, *Passiflora alata* Curtis has emerged as a herbal medicine with anxiolytic properties and central nervous system depressant effects [4, 5].

According to the Brazilian Pharmacopoeia, the herbal drug derived from the leaves of *P. alata* is officially recognized for the preparation of infusions, tinctures, and standardized extracts intended for the treatment of anxiety and insomnia [6]. Additionally, classical monographs, such as those from the European Medicines Agency, provide evidence of the safety and efficacy of species within the *Passiflora* genus, supporting their traditional and rational use in the management of sleep and anxiety disorders [7]. In Brazil, pharmacists have legal authorization to prescribe medicinal plants and manage self-limiting health conditions, including cases of anxiety [8, 9]. Although pharmacists do not provide medical diagnoses of anxiety disorders, they can identify signs and symptoms consistent with self-limiting anxiety, such as restlessness, tachycardia, muscle tension, insomnia, and difficulty concentrating, and manage initial care, referring severe cases to a physician in accordance with the guidelines of the Federal Council of Pharmacy and the Pan American Health Organization [10, 11]. Early pharmaceutical care may prevent the chronification of symptoms and the need for more complex treatments. Studies have reported benefits of clinical pharmacy services in both chronic and acute conditions, such as systemic arterial hypertension, diabetes mellitus, dyslipidemia, headache, dysmenorrhea, aphthous stomatitis, pediculosis, diarrhea, constipation, nasal congestion, cough, labial herpes, candidiasis, and anxiety [12–16], although specific evidence regarding the use of *P. alata* in anxiety management remains limited.

Preclinical studies have investigated the anxiolytic and sedative properties of *P. alata*. For instance, evaluations in Wistar rats demonstrated an anxiolytic effect following acute administration of the extract in swimming tests [17]. Additionally, studies in mice indicated that extracts of *P. alata* and *Passiflora edulis* f. *flavicarpa* promote sedative effects and sleep induction, monitored via telemetry [18]. These findings provide preclinical support for the potential therapeutic use of *P. alata*, but clinical evidence in humans remains limited. It is important to note that conducting clinical studies involving pharmaceutical interventions in Brazil is permitted, provided they are approved by a Research Ethics Committee and comply with the regulations of CNS Resolution No. 466/2012 and CFF Resolution No. 585/2013, which govern pharmacists’ clinical responsibilities. Accordingly, the present study was conducted in accordance with the ethical and legal principles governing human research and pharmaceutical clinical practice [10, 19].

Given the high prevalence of anxiety, the wide availability of this herbal medicine, and the lack of updated studies, this research aimed to evaluate the clinical, humanistic, and economic outcomes of *P. alata* extract use in participants with anxiety monitored by pharmacists in a Brazilian municipality.

## Methods

### Study type, setting, and ethical considerations

This is an open-label, non-randomized, before-and-after experimental study conducted in the Primary Health Care (PHC) network of a Brazilian municipality (Alfenas, state of Minas Gerais), with approximately 80,000 inhabitants. The study was carried out between April 2024 and June 2025. Anxiety management took place at the University Pharmacy (FarUni) of a public university, which offers free pharmaceutical clinical services to the community.

The study was registered in the Brazilian Clinical Trials Registry (Protocol RBR-6fhv7 × 5, registered in august 2023) and approved by a Research Ethics Committee (Approval No. 6.116.248, approved in june 2023). The TREND checklist was used for data reporting [20].

### Population, eligibility, and recruitment

Participants aged 18 years or older, reporting symptoms of anxiety according to clinical parameters and the level established by the GAD-7, who were not using psychoactive substances, and who received a prescription for *P. alata* extract following a consultation with a pharmacist at FarUni, were included. Individuals who did not adhere to the treatment, regardless of the reason, were excluded.

Recruitment occurred through spontaneous demand (participants who were aware of and sought the clinical services provided by pharmacists at FarUni for the community), referral (from other healthcare professionals, most commonly nutritionists), and active outreach (through social media and promotion of clinical services at the time of drug dispensing). Additionally, participants were members of the community who used the public healthcare system, regardless of university affiliation.

### Variables and outcomes

The primary outcome was the clinical response, defined as the change in anxiety severity assessed by the Generalized Anxiety Disorder-7 (GAD-7) scale between baseline (t₀) and the end of follow-up (t₃). Secondary outcomes included: (i) safety, evaluated through self-reported adverse events; (ii) treatment satisfaction, measured by the Treatment Satisfaction Questionnaire for Medication (TSQM) applied 15 days after treatment initiation and at t₃; and (iii) economic outcomes, estimated by analyzing direct treatment-related costs and comparing the potential cost reduction achieved with *P. alata* extract to the standard pharmacological therapies provided by the Brazilian Unified Health System.

#### Sociodemographic and clinical characterization

At baseline (t0), data were collected from electronic medical records, including age, gender, educational level, occupation, income, presence of chronic diseases, sleep disorders, use of psychoactive drugs, use of Integrative and Complementary Health Practices (ICHP), and *P. alata* extract treatment details.

#### Clinical outcomes

Effectiveness was assessed using the GAD-7 scale, administered at baseline (t0) and after 90 days of treatment (t3). The GAD-7 consists of seven items scored from 0 to 3, with a total score of up to 21 points. The seven items are: (i) feeling nervous, anxious, or on edge; (ii) being unable to stop or control worrying; (iii) worrying excessively about different things; (iv) having difficulty relaxing; (v) being so restless that it is hard to sit still; (vi) becoming easily annoyed or irritable; and (vii) feeling afraid as if something awful might happen. Anxiety severity is classified as follows: 0–4 (minimal), 5–9 (mild), 10–14 (moderate), and 15–21 (severe) [21, 22]. Safety was monitored through participant self-reports and pharmacist observations, with documentation of any potential adverse events.

#### Humanistic outcome

Treatment satisfaction was assessed using the validated TSQM at t1 (15 days after starting treatment) and t3. The TSQM consists of 14 items across four domains: effectiveness (items 1–3), side effects (4–8), convenience (9–11), and overall satisfaction (12–14). Responses are based on a 5- or 7-point Likert scale, except for question 4 (yes/no). Each domain score ranges from 0 to 100, calculated using authorized software [23, 24].

#### Economic outcome

The economic outcome was assessed by comparing partial direct costs between *P. alata* treatment and conventional treatments provided by the Brazilian Unified Health System, over a 90-day time horizon. Conventional treatment included anxiolytics freely available through the Brazilian Unified Health System, such as sertraline, fluoxetine, amitriptyline, clomipramine, and nortriptyline. Following guidelines [25], the pharmacoeconomic analysis was conducted in three steps:

I) Cost identification: Only direct medical costs (medications and consultations) were included. The comparator group used the most frequently prescribed anxiolytics in the Brazilian Unified Health System, based on a local specialist’s recommendation. In the intervention group, *P. alata* extract was compounded individually by FarUni.
II) Resource quantification: Resource use was estimated based on medical records. In the intervention group, each patient attended four pharmaceutical consultations over 90 days, with standardized *P. alata* extract dosages (500 mg). In the comparator group, the average number of medical consultations was estimated based on expert inputand and both consultations occurred within the same estimated period of the study. The selection of the pharmacological agents and their respective daily doses was based on national guidelines, which recommends Selective Serotonin Reuptake Inhibitors (SSRIs) and tricyclic antidepressants [26, 27].
III) Cost valuation: Monetary values were assigned in Brazilian reais (R$). Consultations were priced according to the public health system Management System for the Table of Procedures, Medications, and Orthotics, Prosthetics, and Mobility Aids [27]. Medication costs were based on municipal invoices. For *P. alata*, costs of raw materials, excipients, packaging, and production were obtained from FarUni’s internal system.

### Patient follow-up

Consultations at FarUni followed a structured process described in the literature [28, 29]. Participants were received by pharmacist-researchers who applied a clinical method (subjective and objective data collection), conducted a pharmacotherapy assessment, and developed a care plan including non-pharmacological and/or pharmacological strategies. *P. alata* extract was prescribed when indicated. All consultations were documented in the electronic medical record following the model Subjective, Objective, Assessment, Plan (SOAP) [28].

### Dosage standardization and compounding of *P. alata* extract

The dosing of *P. alata* in the present study was based on preclinical evidence and available clinical studies on different species, considering the absence of a specific monograph for *P. alata*. According to Fenner [30], daily oral administration of *P. alata* showed effects within 14 days at a dose of 300 mg. However, the European Monograph and the Brazilian Ministry of Health recommend a minimum dose of 500 mg of dry extract, which may reach a maximum of 2000 mg/day of the encapsulated herbal drug extract, divided into up to four administrations [6, 7].

The dosing of *P. alata* was determined based on anxiety severity as assessed by the GAD-7. Participants with mild anxiety received 500 mg/day, those with moderate anxiety received 1000 mg/day divided every 12 h, and participants with severe anxiety started with 1500 to 2000 mg/day (3 to 4 capsules throughout the day), according to clinical pharmacist evaluation. In cases where anxiety levels persisted, participants were referred to a specialist physician. This protocol aimed to allow individualized adjustment based on clinical response.

The pharmaceutical intervention lasted a total of 90 days, with biweekly consultations during the first month and monthly thereafter, totaling four in-person visits per participant. This duration was defined according to guidelines from the Brazilian Ministry of Health for the rational use of phytotherapeutics in anxiety disorders [7].

The study used a commercial dry extract of *P. alata*, provided by Brazilian companies that won public bids, from different batches. The extract was prepared from leaves and standardized to 0.07% total flavonoids (expressed as apigenin), with a concentration of 500 mg per capsule, according to the certificate of analysis provided by the manufacturer.

### Treatment follow-up period

Participants were followed in the study for a period of 90 days, in accordance with the rational use guidelines for phytotherapeutics from the Brazilian Ministry of Health, which recommend a safe duration of up to 12 weeks for anxiolytic herbal medicines [31–33]. After this period, participants were referred to the pharmaceutical care service at FarUni, provided by pharmacists not involved in the research.

### Sample size and statistical analysis

The sample size estimation aimed to determine the minimum number of participants required to ensure statistical significance. The calculation, based on the comparison of two means (t0 and t3) [34], was performed using G*Power software v.3.1.9.7, considering a clinically relevant difference of 6 points on the GAD-7 score, an approximate standard deviation of 4, a significance level of 5%, and a statistical power of 95%, without accounting for potential losses, resulting in 13 participants; the final sample included a total of 30 participants.

Data were entered into Excel^®^ 7.0 and Jamovi v.2.3.28. Analyses were presented as percentages, graphs, and tables. Quantitative and categorical variables were described by means, standard deviations, medians, and absolute and relative frequencies, and analyzed using Student’s *t* test.

## Results

### Sociodemographic and clinical profile of study participants

Although the sample size calculation indicated a minimum requirement of 13 participants to achieve statistical significance, 53 individuals were recruited to compensate for potential losses and increase the study’s analytical power and representativeness. After follow-up, 30 participants completed all planned study stages.

Among the 30 participants, most were aged 18–28 years (62.5%; mean: 27.8; SD: 10.6), and 80% were female. A total of 63.3% (*n* = 19) were students, one participant (3.3%) was unemployed, and 23.3% were formally employed. Of the total, 37.5% worked ≥ 40 h per week. Regarding education, 70% had incomplete higher education, 20% completed high school, 6.7% had completed higher education, and 3.3% had postgraduate studies. Chronic diseases were reported by 34.4%, and 6.7% were polymedicated. Furthermore, 65.6% had never used psychotropic medications. Regarding sleep patterns, 60% reported poor sleep quality, and 13.3% used complementary and integrative practices, specifically acupuncture. Concerning the *P. alata* extract dose, 50% used 1000 mg/day, 16.7% used 500 mg/day, 30% received 1500 mg/day, and 3.3% used 2000 mg/day.

### Clinical outcome

Regarding anxiety classification, most participants at t0 were classified as having severe anxiety, with a reduction in anxiety levels observed at t3 (Table 1).

**Table 1:** Classification of participants with anxiety treated at FarUni according to the GAD-7 scale, 2025 (*n* = 30)

Anxiety classification (GAD-7)*	t0 *n* (%)	cumulative *n* (%) (t0)	t3 *n* (%)	cumulative *n* (%) (t3)
No anxiety	0 (0,0)	0 (0,0)	9 (30,0)	9 (30,0)
Mild anxiety	2 (6,7)	2 (6,7)	8 (26,7)	17 (56,7)
Moderate anxiety	12 (40,0)	14 (46,7)	12 (40,0)	29 (96,7)
Severe anxiety	16 (53,3)	30 (100)	1 (3,3)	30 (100)

* GAD-7: Generalized Anxiety Disorder 7-item scale [14, 15]

One patient was referred for medical evaluation due to lack of improvement in anxiety symptoms.

In the comparative analysis of variables associated with the clinical outcome (Table 2), the dosage variable was the only one that showed a statistically significant association with the level of anxiety.

**Table 2:** Analysis of sociodemographic and clinical variables associated with clinical outcome, 2025

	Anxiety classification by GAD-7				
Variables	Mild anxiety *n* (%)	Moderate anxiety *n* (%)	Severe anxiety *n* (%)	*p* - value	
Age (years)					
18–28	1(3,3)	7 (23,3)	12 (40,0)		0,44
28–38	0(0,0)	4 (13,3)	2 (6,7)		
38–48	0 (0,0)	1 (3,3)	0 (0,0)		
48–58	1(3,3)	2 (6,7)	0 (0,0)		
Gender					
Female	2 (6,7)	9 (30,0)	13 (43,3)		0,86
Male	0 (0,0)	3 (10,0)	2 (6,7)		
Employment status					
Formal employment	1(3,3)	2 (6,7)	4 (13,3)		0,36
Student	1(3,3)	7 (23,3)	11 (36,6)		
Self-employed	0 (0,0)	2 (6,7)	1 (3,3)		
Public servant	0 (0,0)	1 (3,3)	0 (0,0)		
Weekly working hours					
≥ 40 h	0 (0,0)	5 (16,7)	8 (26,7)		0,75
Unemployed	2 (6,7%)	6 (20,0%)	9 (30,0%)		
Education level					
Completed high school	0 (0,0)	4 (13,3%)	5 (16,7)		0,70
Incomplete higher education	2 (6,7%)	5 (16,7%)	10 (33,3%)		
Completed higher education	0 (0,0%)	1 (3,3%)	2 (6,7%)		
Psstgraduate	0 (0,0%)	1 (3,3%)	0 (0,0%)		
Chronic disease					
Yes	1 (3,3)	5 (16,7)	4 (13,3)		0,70
No	1 (3,3)	7 (23,3)	12 (40,0)		
Polypharmacy					
Yes	0 (0,0)	1 (3,3)	1 (3,3)		1,0
No	2 (6,7)	11 (36,6)	16 (53,3)		
Poor sleep quality					
Yes	0 (0,0)	4 (13,3)	5 (16,7)		0,20
No	2 (6,7)	7 (23,3)	12 (40,0)		
Use of integrative and complementary practices					
Yes	1 (3,3%)	1 (3,3%)	2 (6,7%)		0.67
No	1 (3,3%)	10 (33,3%)	15 (50%)		
Dose of *P. alata* (mg)					
500	2 (6,7)	1 (3,3)	1 (3,3)		< 0,001
1000	0 (0,0)	6 (20,0)	6 (20,0)		
1500	0 (0,0)	3 (10,0)	6 (20,0)		
2000	0 (0,0)	1 (3,3)	0 (0,0)		

During follow-up, no serious adverse reactions or clinically significant changes attributable to the use of *P. alata* extract were observed. Only three participants (19.8%) reported mild side effects with no clinical relevance: one case of drowsiness and two cases of transient gastric and intestinal discomfort during the first weeks of use, with no need to discontinue therapy. These findings further reinforce the safety of the phytotherapeutic agent.

### Humanistic outcome

Analysis of the TSQM revealed evidence of improvement in participants’ health condition at t1 (Table 3).

**Table 3:** Treatment satisfaction between t1 and t3 measured by TSQM in participants with anxiety treated at FarUni, 2025 (*n* = 30)

Domains	Category	Mean (sd)	Median (interquartile range)	Min-max values	Shapiro-Wilk test (*p*-value)	Paired t-test (t0 vs. t3 *p*-value)
Effectiveness	*t1*	73,5 (23,3)	77,8 (22,2)	11,0–100	0,85 (< 0,001)	0,46
	*t3*	70,3 (22,0)	77,8 (19,2)	16,7–100	0,89 (0,004)	
Side effects	*t1*	6,0 (19,1)	0 (0,0)	0–82,4	0,36 (< 0,001) 0,32 (< 0,001)	0,90
	*t3*	5,4 (19,1)	0 (0,0)	0–94,1		
Convenience	*t1*	87,3 (11,5)	88,9 (20,8)	66,6–100	0,88 (0,002)	0,06
	*t3*	82,7 (19,4)	89,9 (27,8)	16,7–100	0,82 (< 0,001)	
Global satisfaction	*t1*	85,2 (17,6)	89,3 (21,4)	28,6–100	0,80 (< 0,001)	0,15
	*t3*	81,2 (18,4)	82,2 (14,3)	28,6–100	0,85 (< 0,001)	

### Economic outcome

The following (Table 4) shows the treatment costs over 90 days, highlighting significant diferences between protocols involving medication with pharmaceutical or medical consultations.

**Table 4:** Total cost of medications and consultations during 90 days of treatment

Medication and daily dose	Medication cost for 90 days *r*$ (real)	Consultation cost for 90 days *r*$ (real)	Total cost for 90 days *r*$ (real)
***P. alata*** 500 mg/dia	13,50	25,20*	38,70
***P. alata 1000 mg/dia***	25,50	25,20*	50,70
***P. alata 1500 mg/dia***	37,50	25,20*	62,7
***P. alata 2000 mg/day***	43,30	25,20*	68,50
Sertraline hydrochloridae 50 mg/day	9,00	70,00**	79,00
Sertraline hydrochloridae 100 mg/day	33,30	70,00**	103,30
Sertraline hydrochloridae 150 mg/day	42,30	70,00**	112,30
Sertraline hydrochloridae 200 mg/day	66,60	70,00**	136,60
Fluoxetine hydrochloridae 20 mg/day	3,11	70,00**	73,11
Amitriptyline hydrochloridae 25 mg/day	3,33	70,00**	73,33
Amitriptyline hydrochloridae 50 mg/day	6,66	70,00**	76,66
Clomipramine hydrochloridae 10 mg/day	26,10	70,00**	96,10
Clomipramine hydrochloridae 25 mg/day	76,50	70,00**	146,50
Nortriptyline hydrochloridae 10 mg/day	83,43	70,00**	153,43
Nortriptyline hydrochloridae 25 mg/day	24,30	70,00**	94,30

* Pharmaceutical consultation cost over 90 days

**Medical consultation cost over 90 days [27]

## Discussion

To date, this is the first experimental study to comprehensively assess the clinical, humanistic, and economic outcomes of *P. alata* extract use in participants with anxiety disorders followed by clinical pharmacists in the context of PHC. The scarcity of clinical studies on this species in scientific databases highlights a gap in the literature and reinforces the relevance of this investigation. Given the traditional use of this herbal medicine for decades, especially in managing anxiety symptoms and sleep disorders, the findings presented here contribute to advancing knowledge by demonstrating its effectiveness, safety, and economic feasibility, suggesting potential for its inclusion in municipal essential medicines lists.

The study revealed a predominance of young women, university students, and individuals with complaints of insomnia, a profile previously identified in research conducted in the same setting [35]. The higher prevalence of anxiety among women is well supported by the literature and is attributed to neurobiological, hormonal, and psychosocial factors [36–38]. Among university students, academic adaptation, performance pressure, and uncertainty about the future increase vulnerability to anxiety disorders [37–39].

The reduction in GAD-7 anxiety scores during the use of *P. alata* extract indicates its effectiveness. Although previous clinical studies have primarily evaluated the related species *P. incarnata*, whose anxiolytic efficacy is comparable to benzodiazepines and antidepressants, our study suggests that *P. alata* may produce similar effects, highlighting the need for species-specific investigation. Chemical differences between *P. alata* and *P. incarnata*, including flavonoid profiles and other active metabolites, may influence the magnitude and profile of therapeutic effects, warranting caution when extrapolating results between species [40–42]. The safety profile was also notable, as no serious adverse events were reported, reinforcing the advantages of using phytotherapeutics in self-limiting conditions.

Although few clinical studies have specifically evaluated *P. alata*, preclinical evidence indicates its anxiolytic and sedative effects, as observed in animal models [17, 18]. Clinical studies with related species, such as *P. incarnata*, have shown significant anxiety reduction comparable to benzodiazepines, but with a lower incidence of adverse effects [43–45]. Extrapolation of these findings to *P. alata* is reasonable due to chemical similarities between the species, but it highlights the need for more direct clinical studies to confirm its efficacy and safety. The safety profile observed in the present study, with no serious adverse events, aligns with preclinical literature and suggests a favorable risk–benefit ratio. Additionally, available toxicological data for this species indicate that *P. alata* exhibits low acute and chronic toxicity in animal models, with no significant changes in hepatic or renal parameters, even at high doses [30]. These findings, combined with the absence of serious adverse events in this study, support the safety of the phytotherapeutic when used within recommended doses. In the context of pharmaceutical care in primary health care, this profile favors its use in participants with mild to moderate anxiety.

Regarding the humanistic outcome, the high levels of satisfaction reported during the initial phases of treatment, assessed using the TSQM, may be attributed to perceived clinical improvement and the absence of adverse effects. The slight reduction in satisfaction at the end of follow-up reflects symptom remission and a decreased perceived need for treatment. The literature associates patient satisfaction with better adherence, reduced medication changes, and lower demand for emergency care, positively impacting healthcare quality indicators [24, 43–45].

From an economic perspective, the use of *P. alata* extract at 500 mg/day resulted in lower total costs over 90 days compared to conventional medications for mild anxiety. It is noteworthy that, although most participants had moderate to severe anxiety, the economic analysis of standard treatment was based on national protocols for mild anxiety, providing a conservative estimate of the economic impact, as the management of severe anxiety typically requires multiple drugs, adjusted doses, and more frequent medical follow-up, which tends to increase direct costs. However, it is important to highlight that the costs observed in this study for the phytotherapeutic may be overestimated, as they refer to small-scale, compounded preparations, which generally have higher unit costs. Conversely, at an industrial scale and in larger quantities, the cost of *P. alata* is considerably lower, reinforcing its potential for large-scale cost reduction. In addition, the use of *P. alata* extract avoids expenses related to managing adverse effects, which are common with antidepressants and antipsychotics [46]. Studies indicate that phytotherapeutics can reduce costs within the public health system, although few economic studies specifically address this species [47]. Moreover, pharmaceutical care can reduce the need for multiple consultations or complementary therapies, contributing to more rational use of resources [43, 47]. The prescription of over-the-counter medications, such as *P. alata*, by pharmacists has proven strategic for expanding access, strengthening resolutive care in primary health care, promoting the safe use of phytotherapeutics, reducing the burden on healthcare units, and improving treatment accessibility [2, 14, 32, 36, 48, 49].

Among the limitations of this study is the logistics of supplying raw materials, which involved delays in compounding processes and overpricing of inputs, hindering financial planning and the continuous inclusion of new participants. Additionally, as a non-randomized before-and-after study with no control group, it presents methodological limitations such as selection bias and potential external influences, which limit the generalizability and causal attribution of the results. Nevertheless, the findings provide promising preliminary evidence for the use of *P. alata* extract in anxiety management within PHC, especially when combined with pharmaceutical care. Studies with more robust designs and larger samples are recommended to confirm the clinical benefits, acceptability, and economic viability of the rational use of herbal medicines in public health services.

## Conclusion

Most participants with anxiety followed by pharmacists in PHC and using *P. alata* extract were young women, students, and individuals with no income, presenting physical symptoms and sleep disturbances. The most frequently prescribed dose was 1000 mg/day of dry extract. A significant reduction in anxiety levels was observed at the end of follow-up, with no reports of adverse effects, suggesting the effectiveness and safety of the herbal medicine for mild to moderate cases. Patient satisfaction was high, especially during the first weeks, and the treatment cost was lower than that of conventional medications provided by public health system.

The results indicate favorable clinical, humanistic, and economic outcomes, reinforcing the potential of *P. alata* as a safe and feasible therapeutic alternative in PHC. These findings may inform clinical decisions and support the inclusion of the herbal medicine in municipal essential medicines lists, expanding access to complementary therapies within public health system. Additionally, this strategy may help optimize public spending and reduce the demand for medical visits, thus strengthening pharmaceutical care in PHC. 

## Supplementary Information

Below is the link to the electronic supplementary material.

Supplementary Material 1 (PDF 225 KB)

Supplementary Material 2 (PDF 171 KB)

## Data Availability

The datasets generated and/or analyzed during the current study are available from the corresponding author upon reasonable request.
