# Supplementary material for: Clinical, humanistic, and economic outcomes of Passiflora alata Curtis use in participants with mild to moderate anxiety: a non-randomized experimental study
Source: Daru. 2026 Feb 4;34(1):12. doi: 10.1007/s40199-026-00591-4 (PMC12873022; doi:10.1007/s40199-026-00591-4)
Supplement: Supplementary file 2 — Supplementary Material 2 (PDF 171 KB) [file 40199_2026_591_MOESM2_ESM.pdf]

ANEXO VI- Aprovação da pesquisa no REBEC

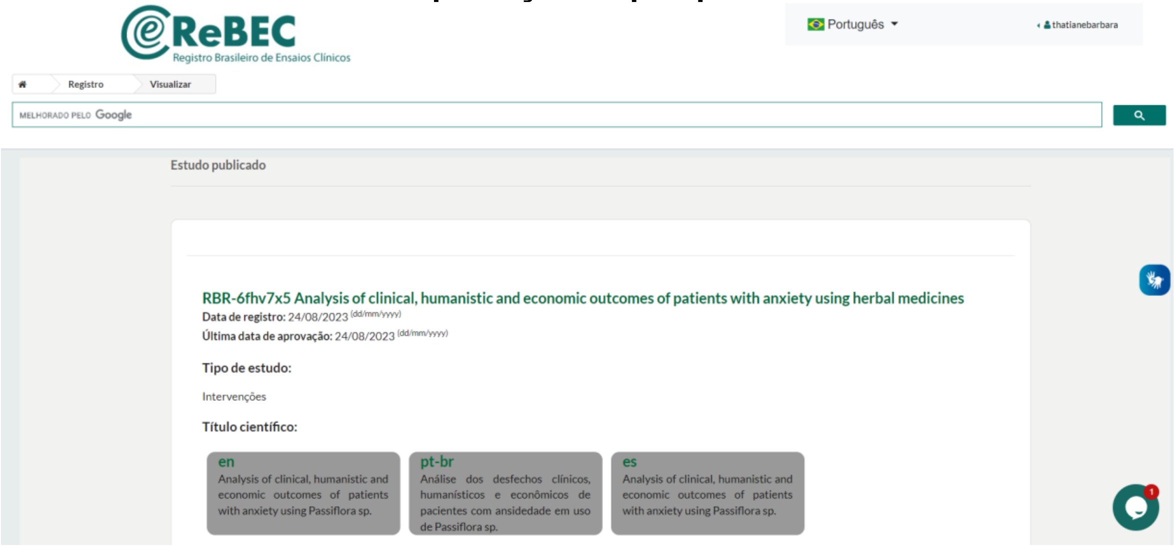
ReBEC
Registro Brasileiro de Ensaios Clínicos
Português
thatianebarbara
Registro › Visualizar
MELHORADO PELO Google

Estudo publicado

RBR-6fhv7x5 Analysis of clinical, humanistic and economic outcomes of patients with anxiety using herbal medicines

Data de registro: 24/08/2023 (dd/mm/yyyy)

Última data de aprovação: 24/08/2023 (dd/mm/yyyy)

Tipo de estudo:

Intervenções

Título científico:

en Analysis of clinical, humanistic and economic outcomes of patients with anxiety using Passiflora sp.

pt-br Análise dos desfechos clínicos, humanísticos e econômicos de pacientes com ansidedade em uso de Passiflora sp.

es Analysis of clinical, humanistic and economic outcomes of patients with anxiety using Passiflora sp.

ANEXO VII

UNIVERSIDADE FEDERAL DE ALFENAS - UNIFAL

COMPROVANTE DE ENVIO DO PROJETO

DADOS DO PROJETO DE PESQUISA

Título da Pesquisa: Análise dos desfechos clínicos, humanísticos e econômicos de pacientes com ansidedade em uso de Passiflora sp.

Pesquisador: Tiago Marques dos Reis

Versão: 2

CAAE: 68446323.0.0000.5142

Instituição Proponente: UNIVERSIDADE FEDERAL DE ALFENAS - UNIFAL-MG

DADOS DO COMPROVANTE

Número do Comprovante: 031433/2023

Patrocionador Principal: Financiamento Próprio

Informamos que o projeto Análise dos desfechos clínicos, humanísticos e econômicos de pacientes com ansidedade em uso de Passiflora sp. que tem como pesquisador responsável Tiago Marques dos Reis, foi recebido para análise ética no CEP Universidade Federal de Alfenas - UNIFAL em 03/04/2023 às 15:13.

Endereço: Rua Gabriel Monteiro da Silva, 700 - Sala O 314 E
Bairro: centro **CEP:** 37.130-001
UF: MG **Município:** ALFENAS
Telefone: (35)3701-9153 **Fax:** (35)3701-9153 **E-mail:** comite.etica@unifal-mg.edu.br
